# The regulation of PBXs and their emerging role in cancer

**DOI:** 10.1111/jcmm.17196

**Published:** 2022-01-23

**Authors:** Ying Liu, Xiang Ao, Xuehao Zhou, Chengcheng Du, Shouxiang Kuang

**Affiliations:** ^1^ Institute for Translational Medicine The Affiliated Hospital of Qingdao University Qingdao Medical College Qingdao University Qingdao China; ^2^ School of Basic Medical Sciences Qingdao Medical College Qingdao University Qingdao China

**Keywords:** biomarker, cancer, PBXs, post‐translational modifications, therapeutic target

## Abstract

Pre‐B‐cell leukaemia transcription factor (PBX) proteins are a subfamily of evolutionarily conserved, atypical homeodomain transcription factors that belong to the superfamily of three amino acid loop extension (TALE) homeodomain proteins. Members of the PBX family play crucial roles in regulating multiple pathophysiological processes, such as the development of organs, congenital cardiac defects and carcinogenesis. The dysregulation of PBXs has been shown to be closely associated with many diseases, particularly cancer. However, the detailed mechanisms of PBX dysregulation in cancer progression are still inconclusive. In this review, we summarize the recent advances in the structures, functions and regulatory mechanisms of PBXs, and discuss their underlying mechanisms in cancer progression. We also highlight the great potential of PBXs as biomarkers for the early diagnosis and prognostic evaluation of cancer as well as their therapeutic applications. The information reviewed here may expand researchers’ understanding of PBXs and could strengthen the clinical implication of PBXs in cancer treatment.

## 1 INTRODUCTION

Cancer is one of the most common diseases and the second leading cause of death in all countries worldwide.^1^ According to data from global cancer statistics in 2020, new cancer cases were estimated to be over 19.3 million and the total number of cancer‐related deaths more than 10.0 million worldwide.^2^ The incidence and mortality of cancer are both rapidly growing, becoming a major barrier to increasing life expectancy. Although some important progress has been made in cancer treatment, it is still a major public health problem worldwide due to the lack of effective therapeutics.^3^ Clinically, a large number of cancer patients are diagnosed at an advanced stage, with a poor prognosis.^4^, ^5^, ^6^ This is mainly caused by difficulties involved in observing the early symptoms of cancer, deficiencies in effective prognostic evaluation methods and limited knowledge regarding the carcinogenesis mechanism. Therefore, it is important to elucidate the underlying mechanism of carcinogenesis, screen novel therapeutic targets of cancer and identify valuable biomarkers for the diagnosis and prognostic assessment.

Pre‐B‐cell leukaemia transcription factor (PBX) proteins are evolutionarily conserved, atypical homeodomain transcription factors that belong to the superfamily of three amino acid loop extension (TALE) homeodomain proteins.^7^ Currently, four members of the PBX family, including PBX1, PBX2, PBX3 and PBX4, have been identified in the human genome. PBX proteins were initially identified as essential Hox cofactors playing crucial roles in early embryonic development and organogenesis^8^ In recent years, an increasing number of studies have suggested that the dysregulation of PBXs is closely associated with many diseases, particularly cancer.^9^, ^10^, ^11^ The high expression of PBXs has been observed in multiple solid tumours, such as prostate cancer (PCa), non‐small‐cell lung carcinoma (NSCLC) and glioma.^12^, ^13^, ^14^ The aberrant expression and/or activity of PBXs contributes to cancer progression by regulating many aspects of cancer cell behaviour, including proliferation, apoptosis, cell cycle, epithelial‐to‐mesenchymal transition (EMT), invasion, metastasis and stemness.^15^, ^16^, ^17^ These characteristics endow PBXs with great potential as therapeutic targets, and biomarkers for early diagnosis and prognostic evaluation in the clinical treatment of cancer patients. Although PBXs have been shown to play crucial roles in various types of cancer, their detailed mechanisms and clinical implications in cancer progression are still unclear.

In this review, we summarize the recent findings in the structures, functions, and regulatory mechanisms of PBXs and focus on their underlying mechanisms in cancer progression. We also highlight the great potential of PBXs as biomarkers for the early diagnosis and prognostic evaluation of cancer and their therapeutic application. Finally, we explore the future research directions of targeting PBXs for cancer therapy.

## 2 OVERVIEW: STRUCTURES AND FUNCTIONS OF PBXS

### 2.1 Structures of PBXs

**FIGURE 1:**
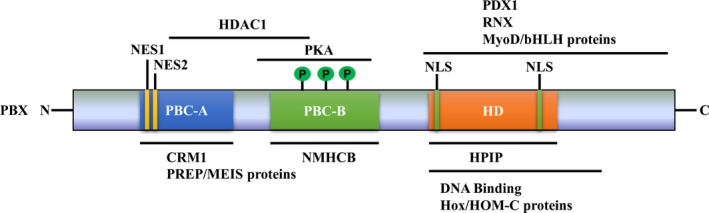
Structure diagram of PBX proteins. PBX proteins share similar conserved structure. PBC‐A and PBC‐B are their family conserved domains, which mediate the interaction of PBXs with other proteins, including HDAC1, PKA, CRM1, NMHCB and PREP/MEIS. HD is primarily responsible for direct interaction between PBXs and DNA, which also mediates their interaction with PDX1, RNX, HPIP, MyoD/bHLH and Hox/HOM‐C proteins. The proteins interacting with PBXs are exhibited above or under the lines at the corresponding regions. PBX, pre‐B‐cell leukaemia transcription factor; NLS: nuclear localization signals; NES: nuclear export sequence; PBC, protein interaction domain; HD, homeodomain; PKA: protein kinase A; HDAC1, histone deacetylase 1; PDX1, pancreatic and duodenal homeobox 1; RNX, radical nephrectomy, MyoD, Myogenic determination gene number; bHLH, basic helix‐loop‐helix protein; CRM1, chromosome region maintenance 1; NMHCA, non‐muscle heavy chain myosin lla; PbxIP1, PBX1‐interacting Protein; OCT‐1, octamer transcription factor 1; FOXC1, Forkhead box C1; PREP, prolyl endopetidase; MEIS, myeloid ecotropic viral insertion site; HOM‐C, homeotic gene complex

The PBX family consists of PBX1, PBX2, PBX3 and PBX4, which share similar conserved structures (Figure 1). PBX1‐3 is about 430 amino acid residues long, while PBX4 is shorter, missing 78 amino acid residues in the N‐terminal domain and a 30‐residue stretch in the C‐terminal domain. PBXs contain a 60‐residue‐long homeodomain (HD) that mediates their binding to DNA or other proteins.^18^ For instance, HPIP competitively bound to the HD domain of PBX1 to inhibit the formation of PBX1‐HOX complexes.^19^ Besides, the HD and C‐terminal of PBX family members mediated their interaction with MyoD and bHLH proteins, thereby inducing muscle differentiation.^20^, ^21^ In another study, PBX1 was found to directly interact with PDX1 via its HD and C‐terminal domain to form a heterodimer, which bound to the promoter of s*omatostatin* gene to activate its transcription.^22^ Furthermore, the HD and C‐terminal of PBX3 mediated its binding to RNX to form a DNA‐binding complex, thereby enhancing the transcription of downstream target genes.^23^ In addition, the HD and C‐terminal of PBX proteins could also mediate their interaction with Hox/HOM‐C proteins to play a role in transcriptional programmes.^24^, ^25^ PBX family members also contain two nuclear localization signals (NLSs) in the HD region and two distinct nuclear export sequence (NES),^26^ which mediates their subcellular distribution by modulating the balance between the import and export pathways. In addition, PBX proteins include two highly conserved protein–protein interaction domains, including PBC‐A and PBC‐B.^27^ The PBC‐A domain of PBX members could mediate their interaction with PREP/MEIS proteins to form transcriptional regulation complexes, which play a role during development.^28^ PBC‐A domain also mediated the interaction of PBX1 with CRM1, which is required for the nuclear export of PBX1, whereas PBC‐B domain in PBX1 could be specifically phosphorylated by PKA, leading to the accumulation of PBX1 in nucleus.^29^ Moreover, the region of amino acids 75–230 (including partial PBC‐A domain and complete PBC‐B domain) in PBX4 was found to mediate the interaction of PBX4 with MEIS and HDAC1, resulting in the reduction of HDAC‐mediated transcriptional inhibition.^30^ In addition, NMHCB competitively interacted with PBC‐B region of PBX1, resulting in the cytoplasmic accumulation of PBX1.^31^

### 2.2 Functions of PBXs

The PBX proteins are key transcription factors of multiple gene regulatory networks and regulate embryonic development, especially the anteroposterior patterning of the main body axis and the limbs by inducing the transcription of target genes involved in cell proliferation, apoptosis and differentiation via interacting with a subset of Hox proteins.^7^ PBXs also play a fundamental and pleiotropic role in the organogenesis of multiple organs, including the heart, lung, thymus, pancreas and spleen.^32^ Moreover, PBXs are strongly associated with cytoskeleton assembly and/or regulation.^31^ In addition, PBXs have been shown to play crucial roles in the processes of haematopoiesis and embryonic stem cell (ESC) pluripotency. For instance, PBX1 is an essential factor for maintaining haematopoietic stem cells and progenitor expansion.^33^ PBXs in the splenic niche also have non‐cell autonomous functions that contribute to the modulation of haematopoiesis partly by targeting KitL/SCF and Cxcl12/SDF‐1.^34^ Furthermore, PBX1 was found to be involved in ESC pluripotency by regulating Fgf8 expression in CCE ES cells.^35^ The complicated functions of PBXs indicate that their dysregulation can lead to various diseases, particularly cancer. Indeed, the aberrant expression of PBXs has been observed in many types of cancer.^12^, ^13^, ^14^ Thus, further studies are required to elucidate the pathological mechanisms and clinical applications of PBXs in cancer.

### 2.3 Molecular mechanisms of PBXs regulation

**FIGURE 2:**
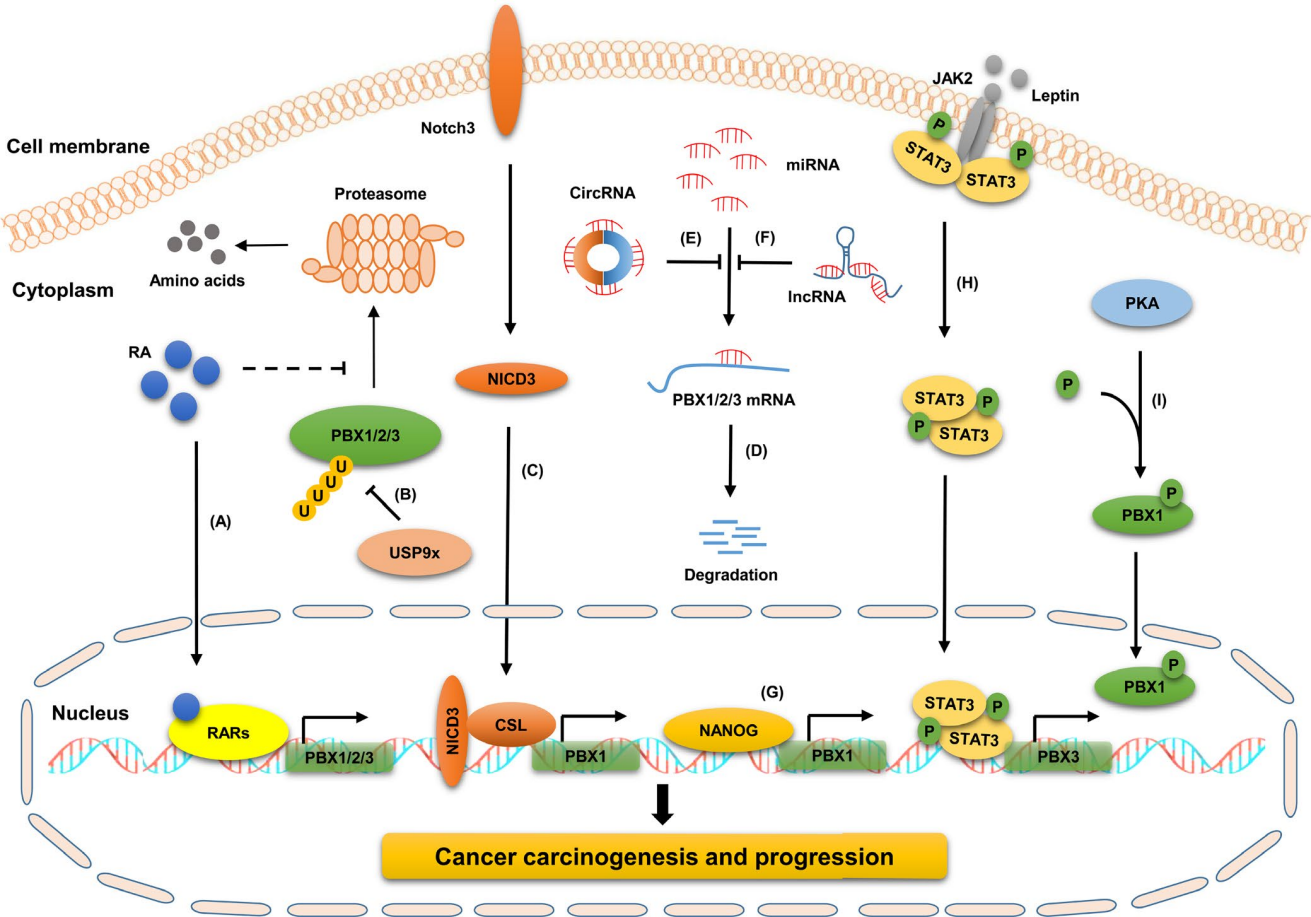
Regulation mechanisms of PBXs. PBXs are regulated by multiple mechanisms at different layers, including transcription, post‐transcription, and post‐translation. (A) RA promotes PBX1/2/3 expression at the transcription level in a RARs‐dependent manner, and inhibits their proteasome‐dependent degradation. (B) USP9x acts as a specific deubiquitinase of PBX1 to inhibit its degradation. (C) NICD3/CSL complex transcriptionally activates PBX1 expression via binding to its promoter. (D) MiRNAs promote the degradation of PBX1/2/3 by targeting the 3′UTR of their mRNAs. (E) CircRNAs and (F) lncRNAs regulate the expression of PBX1/2/3 by sponging miRNAs. (G) NANOG serves as a transcription factor to enhance the transcriptional activity of PBX1. (H) Leptin upregulates PBX3 expression by activating the JAK2/STAT3 signalling pathway. (I) PKA promotes the nuclear import of PBX1 by phosphorylating the conserved serine residues in the PBC‐B domain of PBX1. PBX, pre‐B‐cell leukaemia transcription factor; RA, retinoic acid; RAR, nuclear RA receptor; USP9x, ubiquitin‐specific peptidase 9 X‐linked; NICD3, notch intracellular domain 3; miRNAs, microRNAs; circRNAs, circular RNAs; lncRNAs, long ncRNAs; JAK2/STAT3, janus kinase 2/signal transducer and activator of transcription 3; PKA, protein kinase A

Multiple mechanisms are involved in regulating PBXs expression and activity at different layers, such as transcription, post‐transcription and post‐translation (Figure 2). Here, we exhibit several modes of PBXs regulation with a primary focus on the mechanisms of PBXs regulation in cancers.

#### 2.3.1 Regulation of PBXs at the transcription level

Retinoic acid (RA) is the most active form of vitamin A, and acts as a ligand for nuclear RA receptors (RARs) to regulate embryonic development, differentiation, and growth. It has been reported that the expression of PBXs was significantly upregulated by RA at the transcription level during endodermal and neuronal differentiation of P19 cells.^36^ Another study revealed that *PBX1* was a downstream target gene of the Notch3 signalling pathway. The Notch intracellular domain 3 (NICD3)/CSL complex transcriptionally activated the expression of PBX1 by directly binding to the *PBX1* promoter segment harbouring the CSL‐binding sequence in ovarian cancer (OC) cells.^37^ Moreover, Liu et al. showed that NANOG overexpression significantly enhanced *PBX1* promoter activity in hair follicle (HF)‐derived mesenchymal stem cells (MSCs), leading to the upregulation of PBX1.^38^ In addition, Pang et al. found that PBX3 expression was regulated by leptin at the transcriptional level. Specifically, leptin significantly increased PBX3 mRNA expression by enhancing the activity of the *PBX3* promoter in a STAT3‐dependent manner in breast cancer (BC) cells.^39^ Collectively, these findings indicate that the expression of PBX family members can be regulated by various factors and signalling pathways at transcription level. These upstream pathways apply extra layers of control to the cellular functions of PBX proteins. Thus, identifying upstream regulators of PBXs and elucidating their regulatory mechanisms may provide novel insights for the development of PBX‐based therapeutic strategy.

#### 2.3.2 Post‐transcriptional regulation of PBXs by non‐coding RNAs

Non‐coding RNAs (ncRNAs) are essential regulatory factors that are involved in all pathological and physiological processes by regulating gene expression and chromatin structure.^40^, ^41^, ^42^ According to their size and biogenesis, ncRNAs are divided into different classes, including microRNAs (miRNAs), circular RNAs (circRNAs) and long ncRNAs (lncRNAs).^6^, ^43^, ^44^ In recent years, a large number of studies have shown that ncRNAs contribute to the post‐transcriptional regulation of PBXs (Table 1).

**TABLE 1:** ncRNAs targeting PBXs in cancer

Cancer types	ncRNAs	PBXs	Function of the interaction	References
Pancreatic cancer	miR‐198	PBX1	Overexpression of miR‐198 in pancreatic cancer cells inhibited tumour growth, metastasis, and promoted survival by directly targeting PBX‐1, MSLN, and VCP.	^47^
	miR‐129‐5p	PBX3	Overexpression of miR‐129‐5p overexpression inhibited the proliferation, migration and invasion, and induced apoptosis of pancreatic cancer cells by directly targeting PBX‐3.	^52^
HNSCC	miR‐31‐3p	PBX1	MiR‐31‐3p downregulated PBX1 expression, and low expression of PBX1 was associated with poor prognosis in HNSCC.	
Lung cancer	miR‐1915‐3p	PBX2	MiR‐1915‐3p overexpression inhibited proptosis induced by etoposide through targeting DRG2 and PBX2 via binding to their 3′‐untranslated region.	^49^
	lncRNA UCA1 miR‐144		Overexpression of lncRNA UCA1 significantly promoted lung cancer cell viability, migration, invasion, and cell cycle progression, but promoted cell apoptosis by upregulating PBX3 via sponging miR‐144.	^120^
Acute erythroleukaemia	hsa‐let‐7c‐5p	PBX2	Overexpression of hsa‐let‐7c‐5p induced apoptosis and necrosis in acute erythroleukaemia cells by downregulating PBX2.	^121^
LSCC	circCORO1C let‐7c‐5p	PBX3	CircCORO1C promoted the proliferation, migration, invasion, and in vivo tumorigenesis of LSCC cells by upregulating PBX3 via sponging let‐7c‐5p.	^69^
CRC	miR‐144	PBX3	Overexpression of miR‐144 inhibited the migration and invasion of CRC cells by targeting PBX3.	^53^
	let‐7c	PBX3	Overexpression of let‐7c significantly inhibited cell migration and invasion in CRC cells by targeting PBX3, K‐RAS, and MMP11, as well as tumour growth and metastases in vivo	^119^
OC	lncHCG11 miR‐144‐3p	PBX3	Knockdown of lncHCG11 inhibited the progression of OC cells by downregulating PBX3 via sponging miR‐144‐3p.	^60^
PCa	miR Let‐7d	PBX3	The expression PBX3 was regulated by androgen in PC cells through Let‐7d.	^51^
	lnc HOXA‐AS2 miR‐509‐3p	PBX3	HOXA‐AS2 promoted cell proliferation, migration, invasion, as well as epithelial‐to‐mesenchymal transition (EMT) in PC by upregulating PBX3 via sponging miR‐509‐3p.	^59^
LC	miR‐424 let‐7c miR‐222 miR‐200b	PBX3	PBX3 expression was downregulated in LC cells by MG132 through upregulating miR‐424, let‐7c, miR‐222, and miR‐200b expression via stabilizing Argonuate2.	^104^
GC	miR‐144‐3p	PBX3	MiR‐144‐3p significantly inhibited the proliferation, migration, and invasion of GC cells by directly targeting PBX3.	^89^
	miR‐320a	PBX3	Overexpression of miR‐320a inhibited GC cell proliferation, invasion and migration, and induced apoptosis by directly targeting PBX3.	^54^
Melanoma	miR‐4458	PBX3	MiR‐4458 inhibited cell proliferation and migration, yet induced apoptosis in melanoma by directly targeting PBX3.	^55^
	miR‐320a	PBX3	Overexpression of miR‐320a inhibited cell migration and EMT of melanoma cells by directly targeting PBX3.	^86^
	miR‐495	PBX3	Overexpression of miR‐495 repressed cell proliferation, migration and invasion in melanoma by directly targeting PBX3.	^92^
HCC	miR‐302a	PBX3	Overexpression of miR‐302a inhibited HCC cells proliferation and induced apoptosis by targeting PBX3 and MAP3K2.	^80^
	lnc ANRIL miR‐144	PBX3	ANRIL overexpression promoted cell viability, migration, invasion and inhibited apoptosis of HCC cells by upregulating PBX3 via sponging miR‐144.	^61^
	miR‐33a‐3p	PBX3	Overexpression of miR‐33a‐3p in HCC cells significantly inhibited cell growth, migration and invasion by directly targeting PBX3.	^123^
	miR‐320a	PBX3	MiR‐320a inhibited HCC cell proliferation, migration, and metastasis by directly targeting PBX3.	^124^
	miR‐424 let‐7c miR‐222 miR‐200b	PBX3	Let‐7c, miR‐200b, miR‐222 and miR‐424 synergistically target PBX3 in HCC tumour‐initiating cells.	^103^
Glioma	miR‐320	PBX3	Overexpression of miR‐320 inhibited glioma cells proliferation, and induced cell cycle arrest and apoptosis by suppressing the MAPK signalling pathway via targeting PBX3.	^79^
	miR‐98	PBX3	Overexpression of miR‐98 inhibited glioma cell invasion and migration by directly targeting PBX3.	^93^
	lncRNA HOST2 let‐7b	PBX3	Overexpression of HOST2 promoted the growth and invasion of glioma cells by upregulating PBX3 via sponging let‐7b.	^125^
BC	lnc uc.38	PBX1	Overexpression of uc.38 inhibited cell proliferation and induced cell apoptosis in BC by upregulating Bcl‐2 family members via targeting PBX1	^58^
	HEIH miR‐200b	PBX3	Overexpression of HEIH promoted cell viability, inhibited cell apoptosis, migration and invasion in BC cells by upregulating PBX3 via sponging miR‐200b.	^62^
AML	miR‐495	PBX3	Overexpression of miR‐495 inhibited cell viability and promoted apoptosis of human MLL‐rearranged leukemic cells by targeting PBX3 and MEIS1.	^126^

Abbreviations: AML, acute myeloid leukaemia; BC, breast cancer; CRC, colorectal cancer; GC, gastric cancer; HCC, hepatocellular carcinoma; HNSCC, head and neck squamous cell carcinoma; LC, liver cancer; LSCC, laryngeal squamous cell carcinoma; ncRNAs, non‐coding RNAs; OC, ovarian cancer; PBX, pre‐B‐cell leukaemia transcription factor; PCa, prostate cancer.

MicroRNAs are short single‐stranded ncRNA molecules that play critical roles in post‐transcriptional gene regulation by repressing translation and/or promoting mRNA degradation via targeting the 3′‐untranslated region (3′UTR) of messenger RNAs (mRNAs).^45^, ^46^ PBXs are direct targets of miRNAs in multiple malignant tumours. For instance, Muller et al. revealed that miR‐198 significantly downregulated the expression of PBX1 by directly targeting the 3′UTR of its mRNA, resulting in reduced tumour growth, metastasis, and the increased survival of PCa patients.^47^ PBX1 was also found to be a target of miR‐31‐3p in head and neck squamous cell carcinoma (HNSCC).^48^ Xu et al. found that PBX2 was a downstream target of miR‐1915‐3p. MiR‐1915–3 directly bound to the 3'UTR of PBX2 mRNA, subsequently downregulating its expression in lung cancer cells.^49^ Moreover, PBX2 was identified as a target of miRNA let‐7c in acute myeloid leukaemia (AML) cells.^50^ PBX3 was also reported to be a direct target of let‐7c, which could then bind to the 3’UTR of PBX3 and inhibit its expression in colorectal cancer.^51^ In addition, PBX3 could also be regulated by a series of miRNAs, such as miR‐144, miR‐129‐5p, miR‐4458 and miR‐320a, in multiple types of cancer.^52^, ^53^, ^54^, ^55^, ^56^ All of these miRNAs have been shown to directly target the 3′UTR of PBX3.

LncRNAs are a class of endogenous transcripts longer than 200 nucleotides with a crucial regulatory function. Increasing amounts of evidence have demonstrated that lncRNAs play crucial roles in cancer progression by acting as endogenous miRNA sponges regulating the expression of genes.^57^ Additionally, PBXs can be regulated by lncRNAs. For instance, Zhang et al. revealed that PBX1 was a target of lncRNA uc.38, and overexpression of uc.38 inhibited the proliferation of BC cells and induced cell apoptosis by repressing PBX1 expression.^58^ Xiao et al. showed that lncRNA HOXA‐AS2 upregulated the expression of PBX3 by sponging miR‐509‐3p to promote PCa progression.^59^ Li et al. found that lncRNA HCG11 promotes the viability of OC cells by inhibiting the binding of miR‐144‐3p to PBX3.^60^ Additionally, PBX3 can also be regulated by multiple lncRNAs, such as HNF1A‐AS1, HEIH, ANRIL and HOXA11‐AS.^61^, ^62^, ^63^, ^64^

Circular RNAs refer to a class of regulatory RNA molecules with a covalently closed continuous loop. A large amount of evidence has shown that circRNAs play important roles in human health and diseases, including cancer.^65^, ^66^, ^67^ Several studies have demonstrated that circRNAs are essential regulators of PBXs. For instance, Zhao et al. established a ceRNA network based on a circRNA–miRNA–mRNA potential interaction in recurrent implantation failure using public databases. They found that hsa_circ_0038383 might regulate PBX1 and HOXA9 expression by sponging hsa‐miR‐196b‐5p.^68^ Wu et al. showed that circCORO1C was highly expressed in both laryngeal squamous cell carcinoma (LSCC) tissues and cell lines. The overexpression of circCORO1C increased the expression of PBX3 by competitively binding to miRNA let‐7c‐5p, leading to the promotion of LSCC malignant progression.^69^ Lu et al. revealed that PBX3 was a target of miR‐137. CircHECTD1 enhanced the drug resistance of gastric cancer (GC) to Diosbulbin‐B (DB) by upregulating PBX3 expression via sponging miR‐137.^70^ Taken together, these findings suggest that a comprehensive regulatory network of ncRNAs for PBXs exists in cancer progression. The crucial role of PBX proteins in cancer progression makes them attractive therapeutic targets, which means that their regulation by ncRNAs may be useful in both preventing and treating a wide variety of cancers. Therefore, the identification of specific ncRNAs targeting PBXs could be a promising strategy for cancer treatment. In‐depth studies are required to further explore ncRNA regulatory networks and elucidate their exact mechanisms in PBX regulation.

#### 2.3.3 Post‐translational modifications contribute to PBX regulation

Post‐translational modifications (PTMs) are crucial mechanisms that regulate protein fundamental functions, such as subcellular location, stability, DNA‐binding affinity and interaction with other proteins.^71^, ^72^ The common PTMs include phosphorylation, ubiquitination, acetylation, methylation and sumoylation. Some studies suggest that PBXs can be modulated by distinct PTMs. PBX1 is a substrate of protein kinase A (PKA). PKA specifically was found to phosphorylate PBX1 at several conserved serine residues in the PBC‐B domain and induced the nuclear import of PBX1, whereas the dephosphorylation of these residues promoted its nuclear export.^29^ PBX1 can also be modified by ubiquitin, which mediates its degradation in a proteasome‐dependent manner. Liu et al. revealed that ubiquitin‐specific peptidase 9 X‐linked (USP9x) was a specific deubiquitinase of PBX1. USP9x enhanced PBX1 stability by decreasing its Lys‐48‐linked polyubiquitination in PCa cells.^12^ In addition, bioinformatics analysis showed that multiple types of PTM sites, such as acetylation, methylation and O‐Glycosylation, also exist in the protein sequence of PBXs, indicating that PBXs are potential substrates of these PTMs. Taken together, these findings suggest that the functions of PBX proteins, such as subcellular distribution and stability, are modulated by a complex array of PTMs via different enzymatic reactions. This means that the dysregulation of PBX induced by PTM system disorder is probably to be a key mechanism driving cancer carcinogenesis and progression. Therefore, elucidating the regulatory mechanism of PTMs on PBXs may provide novel insights for the screening and synthesis of specific drugs targeting PBXs and the designing of PBX‐based strategies for cancer treatment.

## 3 IMPLICATION OF PBXS IN CANCER PROGRESSION

The dysregulation of PBXs is closely associated with many diseases, particularly cancer.^9^, ^10^, ^11^ It has been reported that the aberrant expression and activity of PBXs contribute to cancer progression by regulating gene regulatory networks involved in distinct cellular processes, including cell proliferation, apoptosis, cell cycle, EMT, invasion and metastasis as well as the stemness of cancer cells^15^, ^16^, ^17^ (Table 2). Thus, a comprehensive understanding of PBXs in cancer occurrence and development will provide novel insights for early diagnosis, prognostic evaluation and the design of treatment strategies for cancer patients.

**TABLE 2:** Roles of PBXs in different types of cancer

Cancer types	PBXs	Key message(s)	References
BC	PBX1	PBX1 acted as a pioneer factor to guide ERα to unique genomic regions and PBX1‐dependent transcriptional programme is associated with poor‐outcome in BC patients.	^127^
		Upregulation of PBX1 in aggressive BC was partly mediated by genomic amplification of the PBX1 locu, and was closely associated with metastatic progression in ERα‐positive BC patients.	^128^
	PBX3	PBX3 expression was upregulated by leptin in a STAT3‐dependent manner. Moreover, overexpression of PBX3 in BC cells enhanced letrozole resistance by activating the FGFR1 signalling via interacting with MTA1‐HDAC2 complex.	^39^
ESCC	PBX1	Knockdown of PBX1 enhanced radiosensitivity in ESCC cells and xenografts by activating the STAT3 signalling pathway.	^75^
	PBX2	Low PBX2 expression was associated with better prognosis in ESCC patients. Knockdown of PBX2 inhibited tumour growth and induced apoptosis of ESCC cells by decreasing Bcl‐2 expression.	^85^
GC	PBX1	Overexpression of PBX1 promoted the proliferation and metastasis of GC cells by enhancing EMT process. The Phe252 residue in the first helix of the TALE homeodomain of PBX1 mediated its interaction with HOX.	^87^
	PBX2	PBX2 promoted the proliferation, migration, and invasion of GC cells via cooperating with HOXA6.	^91^
	PBX3	Overexpression of PBX3 promoted the invasion and metastasis of GC cell by facilitating EMT process, possibly via the AKT signalling pathway.	^83^
NSCLC	PBX1	PBX1 inhibited the proliferation of NSCLC cells and increased the phosphorylation of histone H3. Knockdown of PBX1 promoted the proliferation of NSCLC cells.	^129^
	PBX2	The expression of PBX2 was associated with prognosis in NSCLC. Knockdown of PBX2 decreased VCM expression in NSCLC cells.	^13^
PCa	PBX1	Overexpression of PBX1 promoted cell proliferation and enhanced the resistance of PCa cells to doxorubicin and cisplatin. USP9x was a PBX1‐specific deubiquitinase and, which promoted the degradation of PBX1 by increasing its Lys‐48‐linked polyubiquitination.	^12^
	PBX3	Androgen decreased the expression of PBX2 by upregulating Let‐7d in PCa cells.	^51^
AML	PBX2	The expression of PBX2 was regulated by let‐7c and might contribute to the AML phenotype.	^50^
	PBX3	High expression of PBX3 was associated with poor prognosis in AML patients. PBX3 knockdown improved the survival of leukemic mice and reduced the leukaemia burden via decreasing the capacity of LSCs and promoting LSC apoptosis.	^105^
Glioma	PBX3	Knockdown of PBX3 suppressed proliferation and induced apoptosis in glioma cells, while PBX3 overexpression significantly facilitated glioma cell proliferation. The effect of PBX3 on proliferation depended on its regulation on cell cycle progression.	^14^
HCC	PBX3	PBX3 mediated the effect of miR‐302a on proliferation and apoptosis in HCC cells.	^80^
Melanoma	PBX1	PBX1 was a target of PLZF. Knockdown of PBX1 by PLZF inhibited melanoma cell growth by reducing the expression of HoxB7 target genes, including angiopoietin‐2 and MMP‐9.	^130^
	PBX2	PBX2 formed a dimer with HOXB7, and the dimer decreased the expression of c‐FOS by upregulated miR‐221 and miR‐222 in melanoma cells.	^118^
	PBX3	PBX3 was a target of miR‐495. Knockdown of PBX3 inhibited the proliferation, migration and invasion of melanoma cells.	^92^
LC	PBX3	PBX3 was closely associated with the stemness of hepatoma cells, and its degradation was in ubiquitin‐proteasome system‐independent manner.	^104^
OC	PBX1	High PBX1 expression was associated with shorter survival in post‐chemotherapy OC patients. Overexpression of PBX1 promoted CSC‐like phenotypes and enhanced the resistance of OC cells to platinum by upregulating STAT3 via binding to its promoter.	^76^
	PBX3	PBX3 silencing inhibited the progression of OC cells. PBX3 mediated the effect of HCG11/miR‐144‐3p on OC progression.	^60^
CRC	PBX3	Overexpression of PBX3 promoted the migration and invasion of CRC cells by activating the MAPK signalling pathway.	^78^
LSCC	PBX3	PBX3 promoted the malignant progression by enhancing EMT progress in LSCC. PBX3 mediated the effect of circCORO1C/let‐7c‐5p on the malignant progression of LSCC.	^69^
Acute erythroleukaemia	PBX2	PBX2 was downregulated in acute erythroleukaemia by hsa‐let‐7c‐5.	^121^
Ewing sarcomas	PBX2	Knockdown of GPR64 decreased the expression of PBX2 in Ewing sarcomas cells.	^131^
	PBX3	PBX3 was associated with bad prognosis in Ewing sarcomas	^132^
HNSCC	PBX1	Low expression of PBX1 was closely associated with poor prognosis in HNSCC. PBX1 was a target of miR‐31‐3p.	^48^
Lung cancer	PBX2	PBX2 could mediate the negative regulation of miR‐1915‐3p on apoptosis in lung cancer.	^49^
	PBX3	PBX3 overexpression facilitated the proliferation and metastasis of lung cancer cells. Silencing of PBX3 exhibited an opposite effect. PBX3 was a target of miR‐144.	^120^
ccRCC	PBX1	High expression of PBX1 was associated with poor OS in ccRCC. PBX1 knockdown suppressed ccRCC cell viability, proliferation and cell cycle progression by inactivating the JAK2/STAT3 signalling pathway.	^74^
Oesophageal cancer	PBX1	PBX1 acted as pioneer transcription factor to mediate the binding of FoxC1 to ZEB2 promoter in oesophageal cancer cells.	^88^
Neuroblastoma	PBX1	Overexpression of PBX1 inhibited proliferation and anchorage‐independent growth, and promoted RA‐dependent and ‐independent differentiation in neuroblastoma. Knockdown of PBX1 produced an aggressive growth phenotype and RA resistance.	^110^
Hodgkin lymphoma	PBX1	PBX1 was upregulated in Hodgkin lymphoma and affected the differentiation of Hodgkin lymphoma cells by activating NFIB and TLX2.	^133^
Embryonal carcinoma	PBX1 PBX2 PBX3	The expression of PBX1, PBX2, and PBX3 was upregulated during endodermal and neuronal differentiation of embryonal carcinoma P19 cells in a RAR‐dependent subtype‐unspecific manner following RA treatment.	^36^
Cervical cancer	PBX2	rs2856437 at PBX2 was closely associated with invasive squamous cervical cancer	^134^
	PBX3	Upregulation of PBX3 promoted the proliferation of cervical cancer cells by activating the AKT signalling pathway. High expression of PBX3 was associated with poor prognosis in cervical cancer.	^82^

Abbreviations: AML, acute myeloid leukaemia; BC, breast cancer; ccRCC, clear cell renal clear cell carcinoma; CRC, colorectal cancer; EMT, epithelial‐to‐mesenchymal transition; ESCC, oesophageal squamous cell carcinoma; GC, gastric cancer; HCC, hepatocellular carcinoma; HNSCC, head and neck squamous cell carcinoma; JAK2/STAT3, janus kinase 2/signal transducer and activator of transcription 3; LC, liver cancer; LSCC, laryngeal squamous cell carcinoma; NSCLC, non‐small cell lung carcinoma; OC, ovarian cancer; OS, overall survival; PBX, pre‐B‐cell leukaemia transcription factor; PCa, prostate cancer.

### 3.1 Roles of PBXs in regulating cancer‐related signalling pathways

An increasing amount of evidence has demonstrated that PBXs play crucial roles in cancer progression by modulating cancer‐related signalling pathways, such as the Janus kinase 2/signal transducer and activator of transcription 3 (JAK2/STAT3), mitogen‐activated protein kinase (MAPK), and the phosphatidylinositol 3‐kinase (PI3K)/AKT, signalling pathways. Elucidating the exact mechanisms of PBXs targeting cancer‐related signalling pathways may provide us with new insights into cancer occurrence and development. The aberrant activation of the JAK2/STAT3 signalling pathway has been observed in many types of cancer, and it is closely associated with tumorigenesis, cell proliferation, angiogenesis and the migration of cancer cells.^73^ Wei et al. showed that PBX1 was significantly upregulated in clear cell renal clear cell carcinoma (ccRCC) tissues and its upregulation was closely associated with poor prognosis. Moreover, the knockdown of PBX1 inhibited the cell viability and the proliferation of ccRCC cells by inactivating the STAT3 signalling pathway.^74^ Another study revealed that PBX1 silencing significantly enhanced radiosensitization in oesophageal squamous cell carcinoma (ESCC) cells and xenografts by downregulating the expression of STAT3 and p‐STAT3.^75^ In addition, Jung et al. found that the high expression of PBX1 was associated with poor prognosis in post‐chemotherapy OC patients. The overexpression of PBX1 could enhance the transcription of STAT3 by directly binding to its promoter, thereby promoting cancer stem cell‐like phenotypes and increasing platinum resistance in OC patients.^76^

The MAPK signalling pathway plays a crucial role in regulating fundamental cellular processes, such as differentiation, proliferation and apoptosis. Dysregulation in MAPK cascade is closely associated with the occurrence and development of cancer.^77^ It has been reported that the overexpression of PBX3 activated the MAPK signalling pathway by upregulating p‐ERK1/2 expression, leading to the promotion of CRC cell migration and invasion.^78^ Another study revealed that PBX3 knockdown inhibited the MAPK signalling pathway by downregulating the expression of phosphorylated Raf‐1, p38 and ERK1/2, leading to proliferation inhibition and apoptosis promotion in glioma.^79^ In addition, in hepatocellular carcinoma (HCC) cells, PBX3 was found to mediate the effect of miR‐302A on cell proliferation and apoptosis by targeting the expression of p‐p38, p‐ERK1/2 and p‐JNK.^80^ The PI3K/AKT signalling pathway is a classical carcinogenic pathway and contributes to many aspects of cancer progression, such as cell proliferation, metastasis and drug resistance.^81^ The knockdown of PBX3 in cervical cancer cells was found to inhibit cell proliferation in vitro and tumour size and weight in vivo via the inactivation of the PI3K/AKT signalling pathway via downregulating p‐AKT expression.^82^ In addition, PBX3 was found to promote the invasion and metastasis of GC cells by upregulating p‐AKT expression.^83^ These findings suggest that PBXs might be core regulators in the signalling pathway networks during cancer progression.

### 3.2 The effect of PBXs on cancer proliferation and apoptosis

The ability to maintain proliferation and escape apoptosis is the most important characteristics in cancer cell survival. Complex signalling pathways and gene regulatory networks are involved in these cellular processes. An increasing amount of evidence has shown that PBXs commonly play an oncogenic role in cancer progression by promoting proliferation and/or inhibiting apoptosis. For instance, Liu et al. revealed that the overexpression of PBX1 facilitated the proliferation and induced the apoptosis of GC cells by downregulating LATS2 expression via enhancing the transcription of miR‐650 in Helicobacter pylori (HP)‐associated GC.^84^ Qiu et al. found that PBX2 knockdown promoted the apoptosis of GC and ESCC cells, whereas it did not affect their proliferative activities, indicating that PBX2 might promote tumour growth via the repression of apoptosis in GC and ESCC.^85^ In addition, the knockdown of PBX3 by several miRNAs has been shown to inhibit proliferation and/or induce apoptosis in different cancers, such as liver cancer (LC), melanoma and GC.^54^, ^61^, ^86^

The regulation of PBXs on proliferation and apoptosis may be partially due to their positive modulation of cell cycle progression. It has been reported that PBX1 knockdown in ccRCC cells inhibited cell viability and proliferation by blocking cell cycle progression. Mechanistically, silencing PBX1 downregulated Cyclin D1 expression via decreasing the p‐STAT3 level.^74^ Xu et al. revealed that silencing PBX3 resulted in a significant increase in the percentage of cells in G0/G1 phases by decreasing the expression of CDKs and increasing the expression of CDKis, thereby inhibiting cell proliferation and inducing apoptosis in glioma.^14^ In addition, the knockdown of PBX3 was found to inhibit cell proliferation and lead to cell cycle arrest at the G0/G1 phase in papillary thyroid carcinoma (PTC) cells.^63^ Collectively, these data confirm that PBXs can act as oncoproteins to promote cell proliferation and/or inhibit apoptosis in different types of cancer, at least partially due to their regulation on cell cycle progression, but the detailed mechanisms are still not fully understood. Nonetheless, elucidating their underlying mechanism in cancer proliferation and apoptosis would help to precisely utilize PBXs‐based therapeutics in particular type of cancer.

### 3.3 PBXs and EMT in cancer

Epithelial‐to‐mesenchymal transition is an essential biological process for maintaining the physiological functions of cells and its aberrant activation of EMT endows cancer cells with enhanced invasive characteristics and increased drug resistance. Cells undergoing EMT exhibit decreased epithelial marker (e.g. E‐cadherin) and increased mesenchymal marker (e.g. vimentin and N‐cadherin) levels.^1^ PBXs have been shown to be crucial regulators of EMT during cancer progression. For instance, He et al. showed that PBX1 promoted the carcinogenesis of GC cells through EMT induction. The overexpression of PBX1 significantly downregulated E‐cadherin expression and upregulated N‐cadherin, vimentin and Snail expression, whereas the knockdown of PBX1 reversed the expression of these proteins.^87^ ZEB2 is a well‐studied transcriptional suppressor of E‐cadherin. Zhu et al. revealed that PBX1 acted as a pioneer transcription factor promoting the EMT process by enhancing ZEB2 transcription via recruiting FOXC1 to the promoter of ZEB2.^88^ Lamprecht et al. demonstrated that PBX3 was closely associated with EMT. Silencing PBX3 in colon cancer (CC) cells partially reversed the decreased expression of E‐cadherin induced by Snail, whereas PBX3 knockdown upregulated the expression of E‐cadherin. Interestingly, PBX3 expression was EMT‐dependent. The overexpression of Snail or ZEB1 was found to significantly upregulate PBX3 expression in CC cells.^15^ In GC, the overexpression of PBX3 was found to induce the EMT progress by upregulating N‐cadherin and vimentin expression and downregulating E‐cadherin expression.^83^ In addition, PBX3 can also mediate the inhibitory role of some miRNAs, such as miR‐509‐3P, miR‐144‐3p, miR‐320a and miR‐526b, on EMT in a variety of cancer types, including PCa, GC and melanoma.^59^, ^86^, ^89^ EMT has been shown to be engaged in multiple steps of the metastasis process. It is possible that PBXs exhibit their pro‐metastatic function in cancer cells by aberrantly activating the EMT process. These findings support the hypothesis that PBX members are oncoproteins but the underlying mechanisms involved in the regulation of PBXs on EMT need further elucidation, which may provide new insights for the development of strategies based on targeting PBXs for cancer treatment.

### 3.4 PBXs control invasion and metastasis

Invasion and metastasis are the main causes of cancer‐related death worldwide. Recent studies suggest that PBX family members are crucial regulators of invasion and metastasis during cancer progression. For instance, the overexpression of PBX1 was found to promote the invasion and migration of GC cells by downregulating LATS2 via enhancing miR‐650 transcription.^84^ Valosin‐containing protein (VCP) is closely associated with the invasion and metastasis of cancer cells. It has been reported that, in BC, PCa, and CC cells, PBX1 enhanced VCP transcription via binding its 5'‐flanking region, indicating that PBX1 might be involved in the regulation of cellular invasion and metastasis through VCP in these cancer types.^90^ VCP was also a target of PBX2, with the knockdown of PBX2 decreasing the expression of VCP in NSCLC cells, suggesting the regulatory role of PBX2 in invasion and metastasis in NSCLC.^13^ Moreover, PBX2 overexpression was found to promote cell metastasis via cooperation with HOXA6 in GC cells.^91^ PBX3 is recognized as a crucial regulator in the invasion and metastasis in a variety of cancers. For instance, the overexpression of PBX3 promoted invasion and metastasis in GC by activating the EMT process.^83^ PBX3 could also induce invasion and metastasis in CRC and glioblastoma through activation of the MAPK signalling pathway.^78^ In addition, PBX3 was found to mediate the effects of several miRNAs, such as miR‐144, miR‐495 and miR‐98, on invasion and metastasis in cancer.^53^, ^92^, ^93^ The molecular mechanisms involved in invasion and metastasis are very complicated and have not been fully understood; however, these findings strongly suggest that PBXs are effective targets for preventing cancer invasion and metastasis as well as cancer progression.

### 3.5 PBXs in tumour angiogenesis

Angiogenesis is the process where new capillaries grow from pre‐existing blood vessels, which is crucial for the growth and metastasis of a large number of solid tumours.^94^ PBXs are involved in angiogenesis, but their mechanisms in this process have not yet been clearly elucidated. In Chen et al.’s study, PBX3 was found to be upregulated in PTC, and its knockdown inhibited angiogenesis and tumour growth in a xenograft mice model. Mechanistically, PBX3 promoted PTC angiogenesis by activating the AT1R/VEGFR2 pathway.^63^ Additionally, Wu et al. showed that PBX3 could mediate lnc HNF1A‐AS1‐induced angiogenesis in CC by upregulating OTX1 expression.^63^ Moreover, PBX3 overexpression in GC significantly enhanced the activity of matrix metalloproteinase‐9 (MMP‐9), which is closely associated with angiogenesis.^83^ PBX1 also plays a crucial role in angiogenesis. Silencing PBX1 was found to block angiogenesis induced by bFGF in the chick chorioallantoic membrane (CAM).^95^ In addition, the inactivation of PBX1 in the renal vascular mural cell (VMC) progenitors of mice significantly upregulated PDGFRβ expression, leading to non‐productive angiogenesis.^96^ These findings suggest PBX1 might play a key role in cancer angiogenesis. Currently, the effect of PBX2 and 4 on angiogenesis has not been reported. Taken together, these findings strongly suggest that PBX1 and PBX3 are angiogenesis‐promoting factors. Angiogenesis plays a crucial role in the spreading and establishment of cancer metastasis. Therefore, PBX1 and PBX3 exhibit great potential as targets for the development of pharmacological drugs for anti‐angiogenesis in cancer treatment. Further studies are required to clarify the exact roles and mechanisms of PBXs in cancer angiogenesis.

### 3.6 PBXs modulate the stemness of cancer cells

Cancer stem cells (CSCs) play crucial roles in cancer progression and are recognized as one of the main causes of drug resistance, metastasis, and recurrence in cancer.^97^, ^98^, ^99^ They are also important targets for cancer therapy.^100^, ^101^ Understanding the regulation mechanism of CSCs may provide us with novel insights into the development of cancer therapeutic strategies. Recent studies indicated that PBXs are crucial regulators in CSC stemness. For instance, PBX1 overexpression has been shown to promote the CSC‐like phenotypes of OC cells by regulating their target genes (e.g. MEIS1, RXRB and NOTCH3) involved in stemness, while the knockdown of PBX1 was shown to decrease their stem‐like properties.^76^ Besides, Fang et al. revealed that the overexpression of c‐Kit promoted the expression of PBX1 in OC cells in a raft‐phospho‐PHB^Y259^‐dependent manner, and PBX1 upregulation could mediate the promotion of the c‐Kit/phospho‐prohibitin axis on the stemness of OC cells.^16^ In another study, Wong et al. showed that PBX2 and PBX3 could interact with MESI1 to form a trimeric complex, which play a rate‐limiting role in induction and maintenance of MLL‐mediated stem cell transformation.^102^ Additionally, PBX3 was a key factor for HCC tumour‐initiating cells (TICs) to acquire and maintain their properties. The overexpression of PBX3 could drive a crucial transcriptional program to activate the expression of some genes, including CACNA2D1, EpCAM, SOX2 and NOTCH3, which are all involved in the stemness of HCC TICs.^103^ Furthermore, the expression of PBX3 was downregulated in HCC cells treated with MG132 (a proteasome inhibitor), leading to the inhibition of HCC cell stemness. Mechanistically, MG132 decreased PBX3 expression by upregulating the expression of miR‐424, let‐7c, miR‐222 and miR‐200b via stabilizing Argonaute2.^104^ In addition, the high expression of PBX3 has been observed in the leukaemia stem cells (LSCs) of MLL‐fusion induced leukaemia. In the same study, PBX3 knockdown delayed leukaemia progression by reducing the LSC potential of MLL‐AF9 leukaemia via promoting LSC apoptosis.^105^

### 3.7 Regulation effect of PBXs on chemotherapy sensitivity in cancer

The development of drug resistance is considered to be one of the main causes of chemotherapy failure, cancer recurrence and finally patient death.^106^ In‐depth investigation into the regulatory mechanisms underlying drug resistance may provide a new theoretical basis for the improvement of cancer chemotherapy. PBXs have been reported to participate in the regulation of cancer drug resistance. For instance, Jung et al. revealed that the overexpression of PBX1 enhanced the resistance of OC cells to platinum by activating the JAK2/STAT3 signalling pathway. Consistent with this, the JAK2/STAT3 inhibitor could potently sensitize platinum‐resistant OC cells to carboplatin.^76^ PBX1 was also found to promote the resistance of PCa cells to common anti‐cancer drugs such as doxorubicin and cisplatin. Correspondingly, the knockdown of PBX1 was found to abrogate this resistance.^12^ Li et al. showed that PBX3 could mediate the effect of lncRNA HOXA11‐AS on the cisplatin resistance of nasopharyngeal carcinoma (NPC) cells. PBX3 overexpression was found to reverse the inhibitory effect of HOXA11‐AS knockdown on the resistance of NPC cells to cisplatin.^64^ In addition, PBX3 plays a crucial in promoting the resistance of GC cells to DB. Silencing PBX3 has been shown to reverse the effect of miR‐137 inhibition and circHECTD1 knockdown on DB‐sensitivity in GC.^70^ Collectively, these findings suggest that PBXs play crucial roles in the regulation of chemotherapy resistance. Elucidating PBX‐related mechanisms in chemotherapy resistance may provide new insights into the development of new drugs and the adjustment of treatment strategies for chemoresistant patients.

**FIGURE 3:**
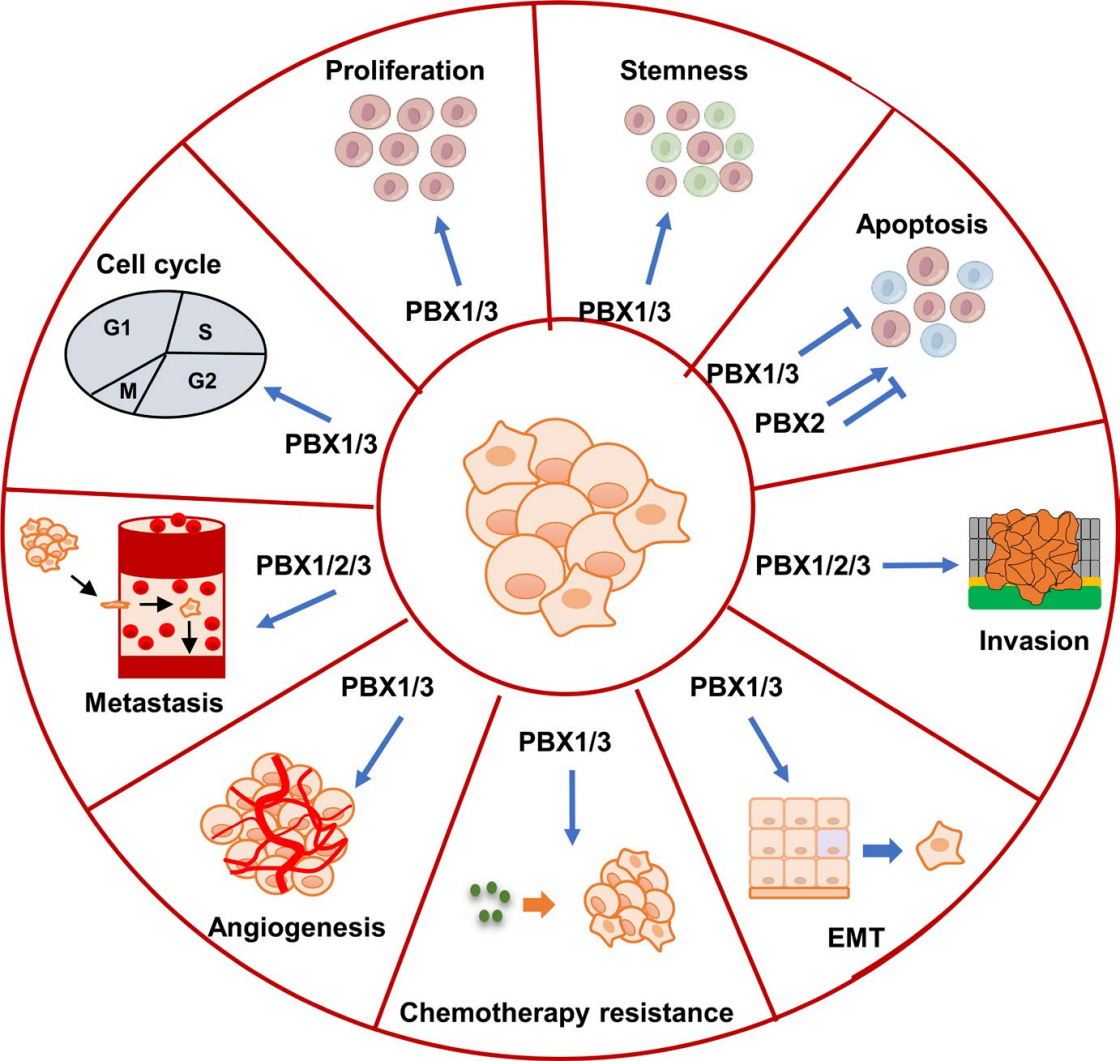
Role of PBXs in cancer progression. PBX1 and PBX3 mainly act as oncoproteins to promote cancer progression by facilitating drug resistance, CSC‐like phenotypes, proliferation, cell cycle, EMT, invasion and metastasis as well as angiogenesis, and by inhibiting apoptosis. PBX2 has dual effects on cancer progression. PBX2 acts as an oncoprotein by promoting invasion and metastasis, and by inducing apoptosis in GC and ESCC. However, in lung cancer, it acts as a tumour suppressor by inducing apoptosis. PBX, pre‐B‐cell leukaemia transcription factor; EMT, epithelial‐to‐mesenchymal transition; GC, gastric cancer; ESCC, oesophageal squamous cell carcinoma

In summary, in most types of cancer, PBX1, 2 and 3 act as an oncoproteins to contribute to cancer progression by promoting drug resistance, CSC‐like phenotypes, proliferation, cell cycle, EMT, invasion and metastasis as well as angiogenesis, and by inhibiting apoptosis. However, PBX2 has dual effects on cancer progression. PBX2 could induce apoptosis in lung cancer, indicating its anti‐tumoural role (Figure 3).

## 4 CLINICAL APPLICATIONS OF PBXS IN CANCER

### 4.1 **PBXs as specific biomarkers during cancer treatment**.

Cancer is a heterogeneous malignant disease with very complicated pathogenesis at the molecular level. The outcome of cancer patients remains poor due to the lack of effective detection methods at an early stage and prognostic evaluation after treatment.^107^, ^108^ Therefore, the identification of new biomarkers with high specificity and sensitivity is expected to greatly contribute to early diagnosis, prognostic evaluation and the adjustment of treatment strategies during cancer treatment.

Accumulating evidence revealed that the aberrant expression patterns of PBXs endow them with great potential as biomarkers for the diagnosis, prognosis and treatment of cancer (Table 3). For instance, our previous work found that the level of PBX1 mRNA was 3.298‐fold elevated in BC tissue compared within adjacent normal tissues in a clinical cohort of 593 specimens from The Cancer Genome Atlas database. The high expression of PBX1 was significantly associated with luminal‐like (*p* < 0.00001) and hormone receptor‐sensitive (*p* = 2 × 10^−5^) subtypes of BC. Moreover, the high expression of PBX1 was shown to be closely associated with poor prognosis in patients with the oestrogen receptor (ER)‐positive (*p* = 0.021), luminal A (*p* = 0.026) and luminal B (*p* = 0.0012) subtypes of BC.^109^ These data strongly suggest that PBX1 is a promising prognostic biomarker for the ER‐positive, luminal A and luminal B subtypes of BC. Besides, Shah et al. evaluated the association of PBX1 expression with prognosis of patients with neuroblastoma (NB) using three independent datasets. In the first dataset consist of 251 NB subjects, they found that the high expression of PBX1 was significantly prognostic of overall survival (OS) (*p* = 0.0004) and event‐free survival (EFS) (*p* = 0.00016). Similar results were obtained in the second dataset consist of 49 NB subjects (OS, *p* = 0.00125) and the third dataset consist of 102 NB subjects (OS, *p* = 0.0002).^110^ These data confirmed that high PBX1 expression is an independently and favourable prognostic biomarker for NB patients. Moreover, PBX1 expression is associated with the sensitivity or resistance of NB cells to retinoid and can be used as a biomarker to predict the response of NB cells to retinoid differentiation therapy.^111^ In another study, Qiu et al. showed that PBX2 was significantly upregulated in gingival squamous cell carcinoma (GSCC) samples, and its high expression was closely associated with poor disease‐free survival (DFS) (*p* < 0.05) and OS (*p* < 0.01). Furthermore, GSCC patients with low PBX2 expression showed a better OS (*p* < 0.05) and DFS (*p* < 0.001) rates compared to those with high expression at poor and moderate differentiations.^112^ These data indicated that PBX2 expression possess great potential to be a new prognostic biomarker for GSCC. Additionally, a ten‐gene panel including PBX2 was identified as a transcriptomic biomarker for distinguishing women with breast lesions from normal controls (sensitivity: 100%, specificity: 84.2%, accuracy: 93.5%, AUC: 0.99).^113^ High expression of PBX3 has been found to be associated with slower progression of patients to castration‐resistant PCa (SHR 0.18, 95% CI: 0.081–0.42, *p* < 0.001). PBX3 expression combined with Gleason score and age showed a high predictive accuracy for diagnosis of PCa patients (SHR 0.21, 95% CI: 0.46–0.93, *p* < 0.001 and AUC = 0.75).^114^ In addition, PBX3 was found to be significantly upregulated in PTC tissues and its upregulation was closely associated poor prognosis of PTC patients (CI: 0.80–44.65; *p* < 0.05),^63^ indicating that PBX3 could act as an independent prognostic biomarker for PTC. Currently, the potential of PBXs as candidate biomarkers in cancer has been validated by many small‐scale studies.^63^, ^109^, ^110^, ^112^, ^113^, ^114^, ^115^, ^116^, ^117^ However, large‐scale studies are still required to further confirm their application as biomarkers in cancer.

**TABLE 3:** PBXs as biomarkers in cancer

PBXs	Cancer types	Function	Clinical values	References
PBX1	BC	Prognostic biomarker	High expression of PBX1 was associated with poor prognosis of patients with ER‐positive, luminal A, and luminal B subtypes of BC.	^109^
	ERα‐positive BC	Prognostic biomarker	ERα‐positive BC patients with PBX1 amplification had a median survival of 30.98 months compared to 113.74 months in luminal BC patients without PBX1 amplification.	^128^
	ER‐negative BC	Prognostic biomarker	Coexpression of PBX1 and EMP2 was associated with poor prognosis in ER‐negative BC.	^116^
	Acute lymphoblastic leukaemia	Diagnostic biomarker	PBX1 was identified as new markers for minimal residual disease detection in acute lymphoblastic leukaemia. PBX1 was differentially expressed in up to 81.4% of acute lymphoblastic leukaemia cases.	^135^
	Neuroblastoma	Prognostic biomarker	PBX1 was associated with histologic neuroblastoma subtypes, with highest expression in benign ganglioneuromas and lowest in high‐risk neuroblastomas. Low PBX1 expression predicted poor prognosis in ganglioneuromas.	^110^
		Predictive biomarker	PBX1 expression served as predictive biomarker in neuroblastoma cells to distinguish between sensitivity and resistance to retinoids.	^111^
PBX2	BC	Diagnostic biomarker	A ten‐gene panel, including PBX2, was identified to differentiate women with breast lesions from normal controls (sensitivity: 100%, specificity: 84.2%, accuracy: 93.5%, AUC: 0.99.	^113^
	GSCC	Prognostic biomarker	High PBX2 expression was associated with poor DFS and OS in GSCC.	^112^
PBX3	PCa	Prognostic biomarker Predictive biomarker	High PBX3 expression was closely associated with slower progression to castration‐resistant PCa (SHR 0.18, 95% CI: 0.081–0.42, *p* values <0.001). PBX3 expression combined with Gleason score and age showed a high predictive accuracy (AUC = 0.82). Patients undergoing radical prostatectomy, with high expression of PBX3 showed improved prostate cancer specific survival compared to patients expressing low levels (SHR 0.21, 95% CI: 0.46–0.93, *p* values < 0.001 and AUC = 0.75).	^114^
	CRC	Prognostic biomarker	The hypermethylation of PBX3 in peripheral blood leukocytes predicted good prognosis of CRC, especially for the UICC stage III CRC patients and CC patients.	^117^
	PTC	Prognostic biomarker	High expression of PBX3 was associated with poor prognosis in PTC patients.	^63^

Abbreviations: AUC, area under the curve; BC, breast cancer; CRC, colorectal cancer; ER, oestrogen receptor; GSCC, gingival squamous cell carcinoma; PBX, pre‐B‐cell leukaemia transcription factor; PCa, prostate cancer; PTC, papillary thyroid carcinoma.

### 4.2 **PBXs as promising therapeutic targets for cancer**.

PBXs play crucial roles in many aspects of cancer progression. The high expression of PBX1 in multiple cancers has been demonstrated to promote cellular proliferation, migration, invasion, and metastasis and inhibit apoptosis through different mechanisms.^12^, ^13^, ^14^, ^63^, ^84^, ^90^ The upregulation of PBX2 was also observed to promote metastasis and suppress apoptosis in GC and ESCC cells,^85^, ^91^ whereas its high expression induced cell apoptosis in lung cancer and melanoma.^49^, ^118^ Moreover, PBX3 overexpression was shown to promote cell proliferation, migration, invasion and metastasis as well as induce apoptosis in GC, glioma, CRC and cervical cancer.^14^, ^82^, ^119^ These findings strongly suggest that PBXs act as oncoproteins to play a key role in promoting progression of a variety of cancers, which endows PBXs with great potential as therapeutic targets for cancer patients. Therefore, the screening or synthesis of novel agents targeting PBXs may provide a new pathway for the clinical treatment of cancer patients. Moreover, PBX proteins have been shown to be regulated by several signalling pathways, such as NOTCH and JAK2/STAT3 signalling pathways at transcription level.^37^, ^39^ This means that identification of novel inhibitors targeting a downstream node of these pathways is also an attractive strategy in cancer treatment. In addition, PBXs are downstream targets of some specific ncRNAs, including miRNAs, circRNAs and lncRNAs,^69^, ^120^, ^121^ thus targeting these ncRNAs may also develop an efficient therapeutic strategy for cancer. However, targeting PBXs in cancer treatment has remained in the pre‐clinical stage of testing due to the lack of effective and specific agents and sufficient data support. Nevertheless, using PBXs as novel targets enhances our insight into the therapeutic strategies of cancer.

## 5 CONCLUSION AND PERSPECTIVE

PBX proteins act as core regulators in gene regulatory networks and play crucial roles in multiple physiological and pathological processes by inducing the transcription of target genes involved in cell proliferation, apoptosis and differentiation. The expression and function of PBXs are tightly controlled by multiple mechanisms at different layers, providing a balanced transcriptional programme to guarantee appropriate differentiation, development, embryogenesis and organogenesis. Therefore, the dysregulation of PBXs contributes to the occurrence and progression of many diseases, particularly cancer. An aberrant expression pattern of PBXs has been observed in different types of cancer, and this aberrant expression was significantly correlated with some pathological characteristics of cancer patients, such as DFS and overall OS, indicating their potential as biomarkers for the diagnosis, prognosis and treatment of cancer. PBXs also exhibit great value as therapeutic targets for a number of cancer types due to their central role in transcriptional regulatory networks. The distinct expression pattern of PBXs in different cancers suggest an interesting possibility for tumour‐tissue‐specific therapeutic strategies. Moreover, PBXs have been shown to regulate the resistance or sensitivity of cancer cells to antitumor drugs, suggesting the feasibility of targeting PBXs for chemotherapy. ERα is the main target of endocrine therapy in BC, and the level of functional ERα is recognized as the hallmark to evaluate the success of endocrine therapy.^122^ PBX1 has been shown to act as a pioneer factor to regulate the expression of ERα at the transcription level, indicating the potential role of PBX1 in evaluating the availability of endocrine therapy for BC patients. PBXs have been shown to be direct or indirect targets of some ncRNAs. Therefore, targeting these ncRNAs may provide a new choice in the clinical treatment of cancer patients. However, some challenges in targeting PBXs in cancer treatment still exist due to their crucial roles in maintaining the normal physiological function of the body. Currently, our understanding of PBXs in cancer progression is still insufficient. More extensive studies are required to elucidate the detailed mechanisms of PBXs in regulating cancer progression, and to develop efficient PBX‐based therapeutic strategies in the clinic.

## CONFLICT OF INTEREST

The authors declare that they have no competing interests.

## AUTHOR CONTRIBUTIONS

**Ying Liu:** Conceptualization (lead); Data curation (lead); Formal analysis (lead). **Xiang Ao:** Data curation (equal); Funding acquisition (lead). **Xuehao Zhou:** Data curation (equal). **Chengcheng Du:** Data curation (equal). **Shouxiang Kuang:** Data curation (equal).

## Data Availability

Data sharing not applicable to this article as no data sets were generated or analysed during the current study.
